# Editorial: Insights in hypertension: 2021

**DOI:** 10.3389/fcvm.2022.1003939

**Published:** 2022-09-09

**Authors:** Michel Burnier

**Affiliations:** ^1^Service of Nephrology and Hypertension, Lausanne University Hospital, Lausanne, Switzerland; ^2^University of Lausanne, Lausanne, Switzerland; ^3^Hypertension Research Foundation, St-Légier, Switzerland

**Keywords:** systolic blood pressure, arterial stiffness, pathophysiology, masked hypertension, cognitive dysfunction, polygenic risk score, lipid accumulation product index

Decade after decade, worldwide epidemiological surveys have reported that an elevated blood pressure (BP) is a leading risk factor for the development of cardiovascular and renal diseases in populations. Although both elevated systolic and diastolic BPs have been shown to predict adverse cardiovascular and renal outcomes, the concept of BP attributable risk has changed with time from a “diastolic only” in the 1960's to an almost exclusive focus on systolic BP 40 years later. In this respect, recent analyses have confirmed that both systolic and diastolic hypertension independently predicted adverse outcomes but with a significantly greater effect of systolic hypertension (1).

With the increasing role attributed to systolic BP, new Research Topics linked to systolic BP have been initiated. Their main goals were to assess whether other parameters such as pulse pressure or pulse wave velocity, reflecting arterial stiffness, could represent better predictive factors of the risk associated with an elevated systolic BP because they integrate various components of the pathophysiology of hypertension. Among the *Insights in Hypertension 2021* Research Topics, several papers have addressed these new aspects of hypertension research focusing on the change in disease burden associated with an elevated systolic BP during the last 20 years, on the association between arterial stiffness and incident hypertension and on the use of new simple indices to assess arterial stiffness. The 2021 hypertension Research Topics also present new tools that could be used in basic research as well as in clinical practice, for example to further improve our understanding of the genetic basis of hypertension or to increase the physicians' ability to recognize some important phenotypes of hypertension such as masked hypertension using machine learning approaches. A summary of the six publications included in the *Insights in Hypertension 2021* are presented below.

Chen et al. have used the data from the Global Burden of Diseases, Injuries, and Risk Factors Study 2019 to assess the variations in the global burden and priority diseases attributable to a high systolic BP according to World regions, sex, and age between 1999 and 2019. The main endpoints were age-standardized mortality rate, disability-adjusted life years, years of life lived with disability, and years of life lost according to age, sex, and regions. The database covered 204 countries grouped in 21 areas of the World with various socio-economic levels. As reported previously, the data presented by Chen et al. confirmed the general decline in high systolic BP- associated mortality but with marked discrepancies between high and low socio-economic countries. Indeed, the trend in mortality decreases in high income (but with a recent flattening of the improvement) and rather increases in low socio-economic areas. However, high systolic BP-associated disabilities were not reduced at the global level. Among cardiovascular and renal diseases associated with a high systolic BP, hypertensive heart disease, ischemic heart disease, stroke, and chronic kidney disease (CKD) constituted the greatest burden linked to a high systolic BP. In the case of CKD, the age-standardized mortality rate is actually increasing due to several factors including age, obesity, and diabetes. The findings of this article further emphasize the need to develop new preventive and therapeutic strategies to reduce the clinical burden associated with an elevated systolic BP. However, future strategies need to be adapted to the local conditions in order to increase their efficacy.

The progressive stiffening of arteries is one of the hallmarks of arterial hypertension. Thus, arterial stiffness is an independent determinant of cardiovascular complications and mortality in hypertensive patients. Whether arterial stiffness is only a consequence of the BP elevation or a cause of hypertension remains controversial. In the present series of articles, Saz-Lara et al. present the results of their systematic review and meta-analysis, assessing whether arterial stiffness is associated with incident hypertension, defined as the first occurrence of a systolic BP > 140 mmHg, a diastolic BP > 90 mmHg, or the need to take an antihypertensive therapy. Twelve prospective longitudinal studies for a total of 66,180 normotensive subjects with a follow-up ranging between 2 and 10 years were included in their analyses. Arterial stiffness was assessed using systolic and diastolic BP and pulse wave velocity. In this population of normotensive subjects, 31% developed hypertension during the follow-up. All three measures of arterial stiffness were associated with an increased risk estimate of incident hypertension but with a large heterogeneity of estimates. These results would support a causal role of arterial stiffness in the development of hypertension. However, whether it is one initial mechanism of hypertension or not remains to be demonstrated.

The study by Saz-Lara et al. also suggests that measuring systolic and diastolic BP is as effective as measuring pulse wave velocity in estimating the risk of incident hypertension. This is useful information for general practitioners because a small number of physicians actually measure pulse wave velocity. In this respect, other indices have been proposed. One of them is the Lipid Accumulation Product (LAP) index. So far, this index, which is calculated based on waist to hip circumference and plasma triglycerides, has been studied mainly in relation to obesity and in patients with metabolic risk factors and there was no consistent results regarding the association with pulse wave velocity. In the study by Shi et al., the association between the LAP index and brachial-ankle pulse wave velocity was investigated in a large group of 4,926 Chinese hypertensive patients. A significant positive association between the LAP index and brachial-ankle pulse wave velocity was observed both in the multivariate linear regression and in the multivariate logistic regression analyses with no difference between men and women. However, there was an interaction with diastolic BP, the association being significant only in patients with a diastolic BP < 99 mmHg. Whether LAP will be used as an indicator of arterial stiffness remains uncertain at this stage.

Both age and hypertension are major risk factors for the development of cognitive dysfunction and dementia. In this context, arterial stiffness has also been reported to be a sensitive predictor of cognitive impairment in humans. Yet, the precise mechanisms linking hypertension and the development of cognitive impairment are incompletely understood. In the study by Li et al., authors explored the cognitive status of elderly patients with hypertension in relation to baseline magnetic resonance imaging (MRI) data of hippocampus and amygdala volume and temporal polar cortex thickness. Not surprisingly, hypertensive patients had a poorer overall cognitive status and poorer executive function status than normotensive controls. Interestingly, the cortical thickness of the right temporal pole was significantly greater in hypertensive subjects than in controls, and a negative correlation between the cortical thickness of the right temporal pole and MMSE score at follow-up suggested that hypertension might affect cognitive function by affecting right temporal pole cortical thickness. These results contrast with a previous observation that reported an atrophy of the medial temporal lobe in elderly patients, 70% of them being hypertensive with memory disorders (2). However, the study by Li et al. had a short follow-up and MRI data were obtained only at baseline and not during the course of the development of cognitive dysfunction. Thus, one cannot exclude that morphological changes go through different stages. Nevertheless, these observations confirm that hypertension and an elevated arterial stiffness leads both to functional and morphological changes of brain structures.

Masked hypertension (MH) and masked uncontrolled hypertension (MUCH) are two hypertensive phenotypes referring to the clinical situation of a normal BP in the presence of the physician or a healthcare provider, but an elevated BP out of the office. MH/MUCH are associated with a high cardiovascular risk comparable to untreated hypertension. Clinically, this is a difficult situation for practitioners because the prescription of additional out-of-office BP measurements when office BP is under control is justified only in the presence of unexplained hypertensive complications, such as left ventricular hypertrophy or proteinuria. However, these latter complications are not monitored on a regular basis. Therefore, it would be helpful to have predictive tools enabling the identification of patients with a high risk of having MH or MUCH. Hung et al. have used the data of two cohorts of hypertensive patients from Taiwan, representing 1,386 patients, to develop machine learning-based prediction models to predict the two patterns of masked hypertension. In brief, they considered up to 33 clinical characteristics as candidate variables to develop models based on logistic regression, random forest, eXtreme Gradient Boosting, and artificial neural network. Interestingly, all models featured a high sensitivity and high negative predictive values regarding masked hypertension. However, the random forest model, composed of six predictor variables, had the best overall performance. This model was based on office systolic, diastolic and mean BP, pulse pressure, HDL cholesterol and the use of beta-blockers. With this model, the negative predictive value was 1.0 and the positive predictive value was around 0.6. This study indicates that an early detection of MH and MUCH might be possible based on simple clinical parameters thereby enabling a reduction in the prevalence of this high cardiovascular risk situation.

Hypertension is a complex multifactorial disease with a pathophysiology that is still far from being completely understood. Genome wide associations studies have contributed to the identification of numerous loci associated with BP but all these loci have a modest effect on BP. The polygenic risk score (PRS) represents a weighted sum of the number of risk alleles carried by each individual. Thus, it enables information to be gathered across loci and is more powerful for corroborating the presence of a highly polygenic genetic signal for hypertension. In the last publication of the *Insights in Hypertension 2021*, Maj et al. have applied pathway-specific PRS models to a target cohort of Italian patients and a European validation cohort in order to investigate further specific predictive biomarkers contributing to the underlying hypertension pathology. A strong association between PRS based on UK Biobank data and the hypertension status was detected in the two independent case/control cohorts confirming the presence of strong polygenic components in hypertension. The analysis of pathways characterized by a prominent genetic association with hypertension confirmed some candidates such as the calcium signaling and phosphatidylinositol/inositol phosphate pathways but also identified new candidates such as the VEGF pathway, focal adhesion molecules, the mTOR complex and surprisingly an apparently hypertension-unrelated pathway, i.e., Huntington's disease. These findings open new avenues for potential pharmacological prevention targeting several pathologies related to hypertension.

Thus, taken together, the six publications of the *Insight in Hypertension 2021* provide interesting new information that deserve some attention. I hope that physicians as well as hypertension specialists will appreciate reading these publications.

## Author contributions

All authors listed have made a substantial, direct, and intellectual contribution to the work and approved it for publication.

## Conflict of interest

The author declares that the research was conducted in the absence of any commercial or financial relationships that could be construed as a potential conflict of interest.

## Publisher's note

All claims expressed in this article are solely those of the authors and do not necessarily represent those of their affiliated organizations, or those of the publisher, the editors and the reviewers. Any product that may be evaluated in this article, or claim that may be made by its manufacturer, is not guaranteed or endorsed by the publisher.

## References

[1] Flint AC Conell C Ren X Banki NM Chan SL Rao VA. Effect of systolic and diastolic blood pressure on cardiovascular outcomes. N Engl J Med. (2019) 381:243–51. 10.1056/NEJMoa1803180 31314968

[2] Lilamand M Vidal JS Plichart M De Jong LW Duron E Hanon O. Arterial stiffness and medial temporal lobe atrophy in elders with memory disorders. J Hypertens. (2016) 34:1331–7. 10.1097/HJH.0000000000000954 27136311

